# Altered transcriptional responses in the lungs of aged mice after influenza infection

**DOI:** 10.1186/s12979-022-00286-9

**Published:** 2022-06-01

**Authors:** Ana M. Hernandez, Jim A. Mossman, Franklin R. Toapanta, Dana M. Previte, Ted M. Ross, Gerard J. Nau

**Affiliations:** 1 Division of Infectious Diseases, The Warren Alpert School of Medicine of Brown University, Providence, Rhode Island, USA; 2 Department of Ecology, Evolution and Organismal Biology, Brown University, Providence, Rhode Island, USA; 3 Aspen Neuroscience, 10835 Road to the Cure, San Diego, CA USA; 4 Department of Medicine, University of Maryland School of Medicine, Baltimore, MD USA; 5 Department of Immunology, University of Pittsburgh School of Medicine, Pittsburgh, PA USA; 6 Center for Vaccines and Immunology, University of Georgia, Athens, GA USA; 7 Rhode Island Hospital, Aldrich 722, 593 Eddy St, Providence, RI 02903 USA

**Keywords:** Influenza, Aging, Immunity, Transcriptome, Host defenses

## Abstract

**Background:**

Influenza causes a serious infection in older individuals who are at the highest risk for mortality from this virus. Changes in the immune system with age are well known. This study used transcriptomic analysis to evaluate how aging specifically affects the functional host response to influenza in the lung. Adult (12–16 weeks) and aged (72–76 weeks) mice were infected with influenza and lungs were processed for RNA analysis.

**Results:**

Older mice demonstrated a delayed anti-viral response on the level of transcription compared to adults, similar to the immunologic responses measured in prior work. The transcriptional differences, however, were evident days before observable differences in the protein responses described previously. The transcriptome response to influenza in aged mice was dominated by immunoglobulin genes and B cell markers compared to adult animals, suggesting immune dysregulation. Despite these differences, both groups of mice had highly similar transcriptional responses involving non-immune genes one day after inoculation and T cell genes during resolution.

**Conclusions:**

These results define a delayed and dysregulated immune response in the lungs of aged mice infected with influenza. The findings implicate B cells and immunoglobulins as markers or mechanisms of immune aging. In addition to discovering new therapeutic targets, the findings underscore the value of transcription studies and network analysis to characterize complex biological processes, and serve as a model to analyze the susceptibility of the elderly to infectious agents.

**Supplementary information:**

The online version contains supplementary material available at 10.1186/s12979-022-00286-9.

## Background

Influenza virus is a significant cause of morbidity and mortality in all age groups, with millions of illnesses, hundreds of thousands of hospitalizations, and tens of thousands of deaths annually in the United States [1, 2]. It is also a global health issue, especially during pandemics, with hundreds of thousands of deaths attributable to influenza annually [3, 4]. The burden of influenza in the Unites States during the 2017–2018 season included 959,000 hospitalizations and nearly 80,000 deaths (CDC, https://www.cdc.gov/flu/about/burden/2017-2018.htm).

Elderly individuals bear a disproportionate burden of illness, hospitalizations, and death due to influenza [2–4]. For example, mortality rates can be 10–20 times greater in individuals more than 75 years old compared to those less than 65 years old [3]. In 2017–2018, individuals 65 years old and older accounted for 90% of all influenza related deaths with a mortality rate 60–100 times higher than those less than 50 years old. Importantly, the aged population has a significantly lower immune responses to seasonal influenza vaccines measured in blood [2, 5, 6], with differences evident on microarray analysis [7]. Therefore, a public health crisis is developing with increasing numbers of elderly individuals who are more susceptible to infection and in whom it is more difficult to prevent disease.

Aging is associated with profound changes in the innate and adaptive immune systems, commonly termed immunosenescence [8, 9]. Thymic involution, for example, is a structural change that reduces the output of T cells in older individuals [9]. Germinal center formation is also altered in aged individuals, contributing to a diminished antibody repertoire [9]. In addition to structural changes, there are also intrinsic changes within particular cell populations. *RAG* gene expression is reduced in older animals, which may contribute to a reduction of pre-B cells [9]. Monocytes and macrophages demonstrate aberrant responses to molecules that activate Toll-like receptors (TLR), while PBMC show mitochondrial dysfunction and defects in anti-viral gene expression [10, 11]. Similarly, granulocytes also show diminished function by a number of measures, including superoxide production and phagocytosis [12]. Older individuals, therefore, are at risk for more serious infection, increased morbidity, and higher mortality rates.

Because of the elderly’s risk from seasonal and pandemic influenza, it is essential to understand the pathophysiologic consequences of increasing age on the course of influenza infection. This information is needed to develop new approaches to vaccinate and treat patients in the older age group. We have previously investigated age-related differences to influenza infection using immunologic and virologic techniques [13]. The results were striking because the content of the immunologic responses was similar between adult animals and aged animals. For example, both groups showed an influx of granulocytes, macrophages, cDCs, and CD40^+^ activated T cells [13]. However, significant differences were seen in the kinetics of the response between the two groups. Aged animals were delayed in their antiviral inflammatory response, resulting in prolonged illness with greater weight loss, worse clinical scores, and delayed viral clearance [13].

Nevertheless, lingering questions remain on the physiological changes that occur with aging and the response to influenza. A number of -omics studies have provided valuable insight into aging or influenza, including the transcriptional profile of the aging lung, lung transcription changes in response to influenza, the gene expression pattern of aging immune cells, and the aged lung epithelium response to influenza in cell culture (e.g. [14–17]). The transcriptional differences between adult and aged animals that have been exposed to influenza, however, is uncharacterized.

Weighted Gene Co-expression Network Analysis (WGCNA) identifies clusters (modules) of co-expressed genes in high dimensionality expression datasets such as RNA microarray or RNA-seq [18]. The underlying gene pairwise adjacency matrix can be further used to identify modules that are significantly associated with a phenotypic trait, or to classify those genes whose expression patterns are consistent with highly connected -hub- positioning. One major advantage of module association analysis over node (gene) association analysis is a relaxed penalty of multiple testing, which is unavoidable when assessing significance at the gene level.

Here, we used WGCNA of transcription data to define how host defenses differ between aged and adult mice infected with influenza. The approach identified modules of co-expressed genes that showed patterns consistent with age-specific responses to influenza infection and age associations. These results show a tight correlation with our prior study of age-related alterations in immunological and cellular responses to influenza infection [13]. The results confirmed our previous findings regarding kinetic differences between the two groups, which also acts as a validation of the ability of WGCNA to identify patterns of gene behavior that are biologically relevant. Additionally, we found elevated levels of host defense proteins and immunoglobulin expression in aged mice. Finally, we found distinct subsets of genes that were involved in the response to infection but were age independent. These results help dissect which aspects of the response to infection are most sensitive to aging.

## Results

### Overall findings

**Fig. 1:**
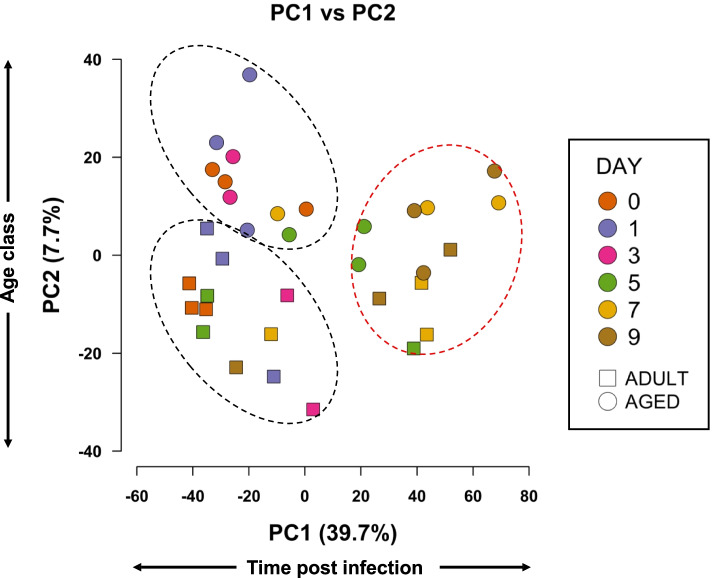
Principal components analysis of global gene expression across two mouse age classes and 6 timepoints (*n* = 34) post influenza infection. The first principal component (PC1; abscissa) explains 39.7% of the variance in gene expression, while PC2 explains 7.7% of the variance. Adult mice are shown as square data; aged mice are represented by circles. Different colors represent different time points post infection. The Euclidean distance between samples reflects their degree of similarity in expression at a global level. Dotted ellipses were manually included to highlight the data structure in the analysis. Early timepoints are highlighted with a black ellipse and later timepoints with a red ellipse

We first explored the global patterns of gene expression among samples using principal components analysis (PCA). PCA revealed that the first component (PC1) explained 39.7% of the variance in gene expression data and was largely associated with the timepoints under study, showing a roughly monotonic change with PC1. The second component (PC2) explained 7.7% of the variance and was associated with the age class of the mice (Fig. 1). Three clusters of data points, defined by age category (adult versus aged) and time after infection, were evident within the PCA plot (Fig. 1). Data points of aged and adult animals on the PCA plot were distinct early in the time series, days 0–3. However, gene expression became more similar later in the time series, days 5–9, when expression in aged and adult animals lost the distinction evident earlier in the time series (Fig. 1).

The gene expression data was next evaluated using WGCNA. WGCNA is a method to study high dimensionality data by using prior expectation that genes do not operate in isolation. Instead, genes that are in the same biochemical pathway or gene–gene interaction network are likely to share correlated expression patterns across genotypes and experimental treatments because of underlying stoichiometry of their protein products. The aim of co-expression network analysis is therefore to identify those sets of genes that are (co)-related in their expression behavior, suggesting they are functionally related. Such genes are described as modular. Modules, too, can be correlated or anti-correlated with each other. Using the module-trait relationship function in WGCNA, we identified a number of statistically supported modules whose expression was consistent with age-associated differences in influenza response after infection of adult (12–16 weeks) and aged (72–76 weeks) mice with mouse-adapted influenza A virus PR8.

In total we identified 95 ‘proper’ modules of co-expressed genes and one ‘improper’ module containing genes with no statistical support of clustering. Supplementary Table S1 describes the gene membership of all modules. The module-trait relationship heatmap (Supplementary Fig. S1) highlights those modules whose eigengene- akin to a ‘first principal component’- is correlated with particular traits. In this analysis the relevant traits were mouse age and day of the treatment (Supplementary Fig. S1). Using this map, we identified a series of modules strongly associated with infection, upregulated in aged mice, and modules shared between age groups after infection.

Next, we formally tested whether any modules demonstrated significant associations between sample eigengene expression and: (a) mouse age class, (b) the shape of expression change over time (spline), and (c) the age x shape interaction term, using *limma* [19]. In the full model, we found statistical support for 15 modules that demonstrated expression patterns different to the null expectation with a False Discovery Rate < 0.05 (FDR < 0.05). Representative gene expression patterns, eigengene values and gene ontology analyses of the 15 statistically supported modules are described in Supplementary Fig. S2. The 15 modules were: ‘salmon4’, ‘purple’, ‘blue’, ‘brown2’, ‘sienna3’, ‘antiquewhite4’, ‘skyblue2’, ‘yellow’, ‘greenyellow’, ‘cyan’, ‘green’, ‘skyblue’, ‘red’, ‘salmon’, and ‘lightcoral’. We also describe an additional module, ‘mediumpurple3’, with strong immune-related gene enrichment and FDR = 0.057. The highlighted modules in Supplementary Fig. S2 show various expression patterns, consistent with the fitted model first-order and interaction terms.

### Select modules demonstrate a lag of immune-related genes in aged mice post-influenza infection

**Fig. 2:**
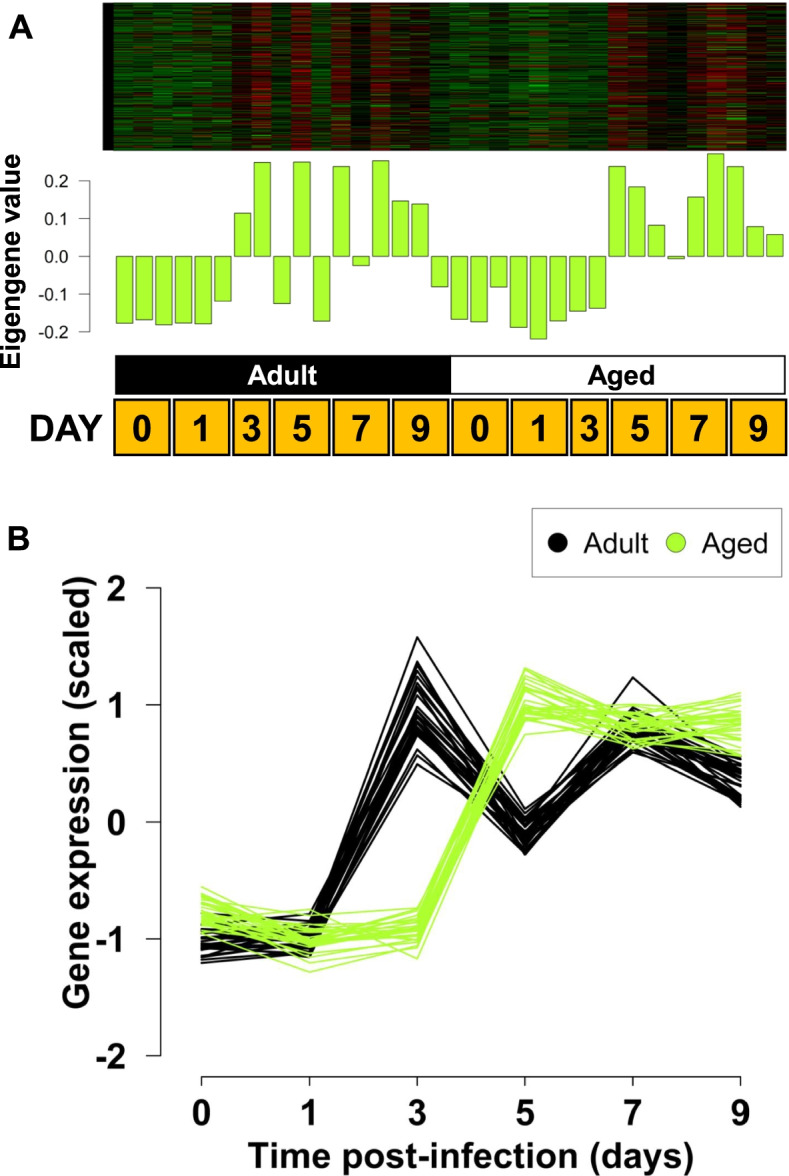
**‘**Greenyellow’ module of co-expressed genes (*n* = 298) demonstrating earlier induction in adult mice than aged mice. Heatmap of scaled gene expression and eigengene barplot (**A**) show adults have generally earlier induction for most genes and samples. Each row of the heatmap represents an individual gene, while each column represents an individual sample. The barplot displays the single eigengene value for that sample, which is essentially a 1.^st^ principal component ‘gene’ of the module. The interaction plot (**B**) shows the behavior of the top 30 genes by their module membership. Each line is a separate gene, and the same genes are shown for both age classes. The samples from adult mice (black lines) are up-regulated one two-day interval earlier than in aged mice (greenyellow lines). A full table of module membership is shown in Supplementary Table S1. The order of timepoints within each age class is: 0, 1, 3, 5, 7, and 9 days post-infection (*n* = 34 total)

We showed previously that immunological responses to influenza were delayed in aged mice [13]. Using WGCNA analysis, one module was identified that showed gene expression responses to influenza that were significantly earlier in onset in adult mice than aged mice. This was the ‘greenyellow’ module (Fig. 2). Expression in the adult animals typically preceded that in aged animals by at least one, two-day interval (Fig. 2B). These gene expression changes, including the difference between adult and aged mice, preceded the protein changes that were described previously [13]. For example, interferon-gamma (IFN-γ) protein was detected on day 5 post infection in adult animals but day 7 post infection in aged animals [13]. Upregulation of *Ifng* expression in the ‘green’ module was noted by day 3 in the adults and day 5 in aged animals. Additional comparison of cytokines and chemokines transcription and protein levels is described below.

Gene ontology analysis was conducted using Gene Ontology enRIchment anaLysis and visuaLizAtion tool (GOrilla) [20]. The genes in the ‘greenyellow’ module revealed enrichment for immune-related ontologies (Supplementary Fig. S2). This module included key immune and anti-viral molecules such as *Ifnb, Ifnl1 (Il28), Il1b, Il6*, acute phase proteins *Saa1,2,3* and numerous interferon-regulated genes including *Mx1, Mx2, Oas1a/b/e/f,* and *Oas2* (“defense response to virus” GO process FDR q-value: 3.83E-36). The ‘green’ module was similar to the ‘greenyellow’ module with a lower response in aged animals on day 3, and contained a broad array of immune genes including complement components, chemokines, *Cd86, Myd88, and Ifng* (“immune system process” GO process FDR q-value: 2.19E-27) (Supplementary Fig. S2).

### Pro-inflammatory cytokines and chemokines are up-regulated earlier in adult mice in response to infection

We next assessed whether pro-inflammatory cytokines and chemokines, which had been previously quantified in a parallel experimental cohort [13], were detected in gene co-expression modules consistent with earlier up-regulation in the younger (adult) mice. For this we focused on the ‘greenyellow’ and ‘green’ modules (Fig. 2 and Supplementary Fig. S2). In these modules, expression increases with experimental time with the adult mice showing an earlier expression response than aged mice. Specifically, the cytokines and chemokines were: IL-1 α (*il1a*), IL-1β (*il1b*), IFN-α (*Ifna1*), IFN-γ (*Ifng*), TNF-α (*Tnf*), IFN-β (*Ifnb1*), IL-12 p70 (*Il12a*), IL-6 (*Il6*), KC (*Cxcl1*), MIP-1β (*Ccl4*), RANTES (*Ccl5*) and MCP-1 (*Ccl2*), IL-12 p40 (*Il12b*). Nine of the 13 gene or protein products assayed in both studies were found in the modules that showed genes up-regulated earlier in the adult mice than in the aged mice: IL-1β (*il1b*), IFN-γ (*Ifng*), TNF-α (*Tnf*), IFN-β (*Ifnb1*), IL-6 (*Il6*), KC (*Cxcl1*), MIP-1β (*Ccl4*), RANTES (*Ccl5*) and MCP-1 (*Ccl2*) (Supplementary Fig. S3). The remaining four genes were found in the module of zero-connectivity (‘grey’ module: *il1a, Ifna1, Il12a*), or an additional significant module (‘salmon’ (FDR < 0.1): *Il12b*). The full list of module membership for each gene in this investigation is shown in Table S2.

### Age-specific modules after infection

**Fig. 3:**
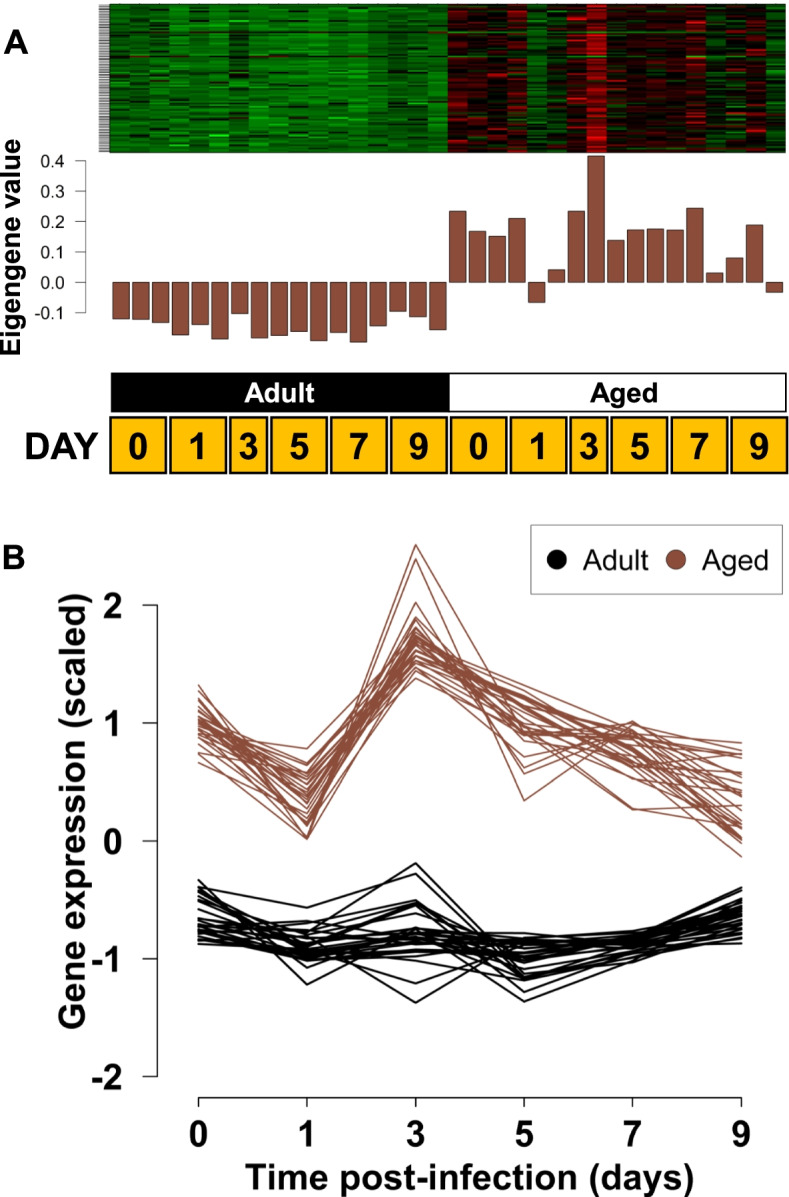
Age-associated module of co-expressed genes. The ‘salmon4’ module demonstrates a clear signal of age-associated co-expressed genes (*n* = 92). The heatmap and barplots (**A**) show the average eigengene expression for each library, which are ordered as increasing timepoints from left to right. Genes are generally down-regulated across all timepoints in adult mice and up-regulated in aged mice. The interaction plot (**B**) shows expression in adult mice is generally stable, while aged mice have systematically higher and less stable expression. The top 30 genes by ranked module membership are shown

Having validated our modules with the results of this earlier publication and confirmed that major cytokines and chemokines are associated with modules that were induced earlier in adult animals, we next tested whether there were modules correlating with differences between the mouse age groups. One module demonstrated a clear signal of age-associated change in gene expression; the module-trait relationship of ‘salmon4’ module (*n* = 92 genes): *r* = 0.88, *p* < 9e-12) (Fig. 3). Genes in this module were principally expressed in aged animals but not in adult animals. GO analysis revealed “immunoglobulin production” as the highest-ranking GO process term (FDR q-value: 8.64e-14), “immunoglobulin receptor binding” as the highest-ranking function term (FDR q-value: 1.69e-05) (Supplementary Fig. S2). The genes comprising this module were dominated by immunoglobulin genes, including kappa, lambda, heavy, and J chains. The ‘blue’ module (FDR < 0.05) expression pattern was similar to ‘salmon4’ and demonstrated similar relatively high expression in aged animals (Supplementary Fig. S2). Additional Ig genes were found in ‘blue’, but also a preponderance of G-protein coupled receptors (GPCRs) associated with olfactory and vomeronasal receptors that can be found in lung tissue [21, 22]. Elevated GPCRs were also observed in the ‘antiquewhite4’ module. The ‘yellow’ module contained genes that increased more in aged animals later in the time series. These genes were strongly associated with cell cycle based on GO analysis (“cell cycle” FDR q-value: 3.02E-57) (Supplementary Fig. S2). This gene list included cell division cycle proteins 1, 2a, 3, 4, 5, 6, 8, 20, and 45, *Brca1* and *Tipin* associated with DNA repair, cyclins E and B1, and oncogenes like *Ski* and *Ect2*. In addition, genes relevant to the immune response were found in ‘yellow’, namely *Il18* and *Cx3cl1*.

Two modules, ‘cyan’ and ‘skyblue2’, were significantly associated with adult animals and anticorrelated with aged animals (Supplementary Fig. S1, S2). GO analysis of these modules, however, failed to identify significantly enriched ontogenies with related biological processes. Modules with similar behaviors were identified on the module-trait relationship figure (Supplementary Fig. S1): ‘lightsteelblue’ and ‘paleturquoise’ with ‘cyan’, and ‘skyblue1’ and ‘white’ with ‘skyblue2’. These additional modules did not meet our FDR threshold and also did not show related biological processes on GO analysis. Nevertheless, genes of immune interest, namely *IFNab* and *IFNz*, were present in ‘paleturquoise’ and ‘cyan’, respectively.

### Responses shared between adult and aged mice

**Fig. 4:**
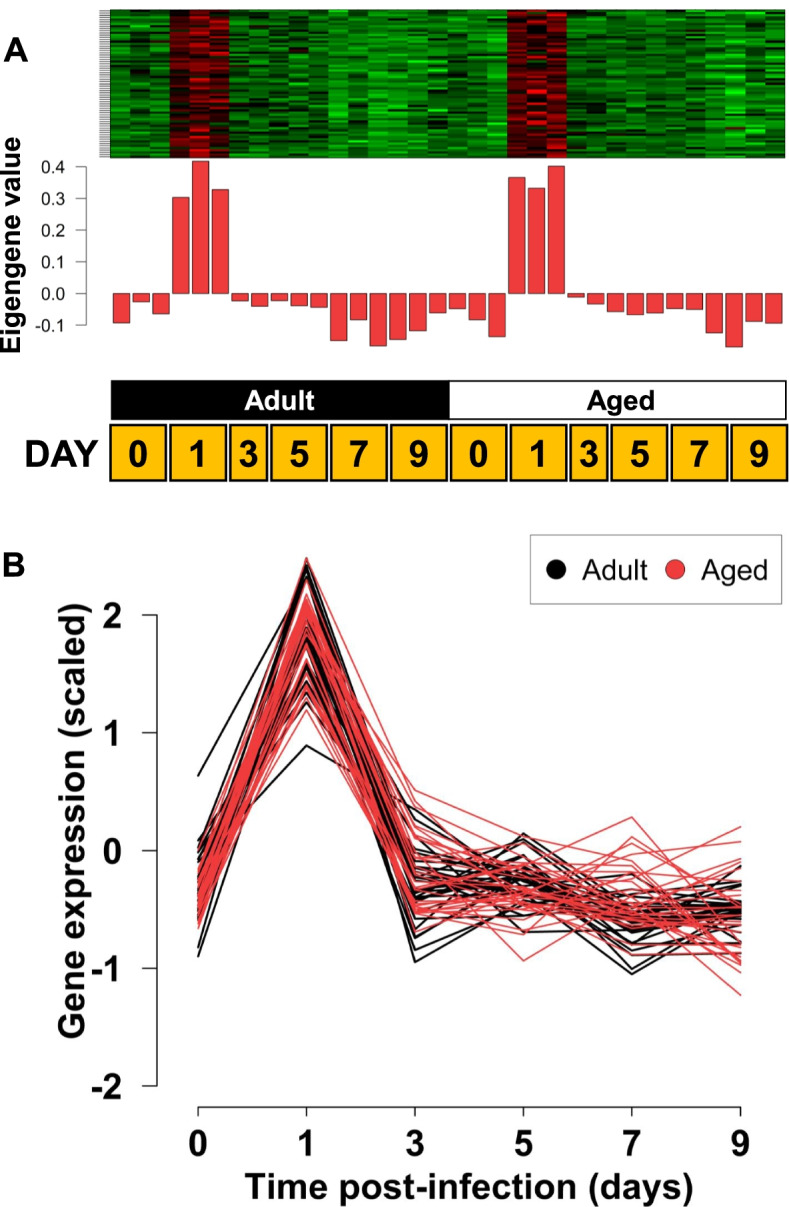
Age independent, immediate post-infection response module. The ‘brown2’ (*n* = 67 genes) module is shown. The heatmap and barplots (**A**) show the average eigengene expression for each sample, which are ordered as increasing timepoints from left to right. Genes were upregulated at day 1 post-infection in both adult and aged mice then returned to pre-treatment levels. Heatmap in (**A**) and interaction plots in (**B**) show consistent response in both mouse age classes and the response is restricted to the Day 1 timepoint post infection. The top 30 genes by module membership are shown

The prior modules showed substantial gene expression differences between the two different age groups, both with a delay in the induction of immune response genes (Fig. 2) and group-specific differences (Fig. 3). This raised the question of whether there were any congruous responses in the animals of different ages. Reviewing the data (Supplementary Fig. S1), the ‘brown2’ module showed genes that were upregulated one day after infection in both adult and aged mice and then returned to pre-treatment levels (FDR = 0.026, Fig. 4). The top genes most correlated with induction one day after infection in the ‘brown2’ module included *Gsta1*, *Uchl1*, *Cbr3, Gsta2* (A_51_P305138 feature), *Gpx2, Srxn1,* and *Acox2*. All of these genes are associated with oxidation/reduction and the response to oxidative stress, consistent with the ‘brown2’ modules ontologies from GOrilla [20] that included glutathione metabolic process (FDR-q value 6.3E-3) and response to oxidative stress (FDR-1 value 2.73E-2). Genes in ‘darkturquoise’, a module with similar behavior to ‘brown2’ early after infection (‘darkturquoise’ FDR = 0.09, Supplementary Fig. S1), also contained genes associated with oxidation/reduction functions including thioredoxin reductase 1 (*Txnrd1*), peroxiredoxin 1 (*Prdx1*), and glutathione reductase (*Gsr*). Multiple chaperonins and related proteins were also found in ‘brown2’ and ‘darkturquoise’, including *Hspd1*, *Hspa5, St13, Sdf2l1*, and *Ero1l*, that was consistent with the GO process ‘de novo’ protein folding (combined modules: FDR q-value: 8.41E-3).

We next sought evidence for similar responses among the adult and aged mice later in the time series. The ‘mediumpurple3’ module was associated with experimental day in both groups of mice (Supplemental Fig. S1 and S2, *r* = 0.68, *p* = 1E-05). GO analysis of this module showed strong enrichment for immune system process (FDR q-value = 4.14E-15) and adaptive immune response (FDR q-value = 1.12E-10). Notable entries in this module included T cell receptor alpha/beta chains, CD3 components (delta, gamma, zeta), T cell receptor signaling molecules like Lck, Zap70, Lat, Prkcq, Skap1 (Scap1), and Txk, and T cell surface markers including Thy1, CD2, CD8a/b, CD5, CD6, and CD27. The finding of T cell genes was supported by a significant enrichment for T cell-related ontologies ‘positive regulation of T cell activation’ FDR q-value = 3.51E-8; and ‘T cell receptor complex’ FDR q-value = 1.8E-7) in the ‘mediumpurple3’ module.

## Discussion

Influenza exacts a heavy toll on aging individuals, with higher rates of morbidity, hospitalization, and mortality on people as they get older [3, 4]. The elderly are less responsive to influenza vaccination, adding to the risk associated with this infection. Understanding the immunological response of aged individuals to infectious challenges is critical to developing targeted interventions for this demographic.

Although aged mice have significantly delayed immunologic responses to influenza compared to adult animals [13], it was not clear how the two groups differed beyond the immunologic parameters that were measured. The objective of this study was to investigate the immunologic and non-immunologic differences between aged and adult animals challenged with influenza virus by transcriptome analysis. The results presented herein confirm and extend the immunology studies showing aged mice lagged in production of key immune proteins such as IL-12, IFN- γ, and IL-6 [13]. In this paper, we used WGCNA to show that the lag in protein production is found using gene expression analysis tools as well. WGCNA clusters genes with similar expression behaviors, which yielded modules demonstrating a lag in transcription responses by the older mice. The expression levels of genes in these modules increased at later time point in the aged mice compared to the adult. The content of these modules was dominated by host defense genes and an anti-viral response (Fig. S2 and Supplementary Table S1). We were able to identify 9 out of 13 proteins from our initial study in one of these “lag response” clusters (Fig. 1). This underscores the power of WGCNA to capture complex gene behavior patterns and validates the results due to the high correlation with the measured immunological responses. The RNA changes preceded the measurable immunological responses, suggesting RNA was an earlier indicator of the immune response.

In addition to a lag in immune response genes, there were substantial differences in genes that were specifically upregulated in older mice. These age-associated modules, ‘salmon4’, and to a lesser extent, ‘blue’, ‘antiquewhite4’, and ‘yellow’, were populated with immunoglobulin genes (Supplementary Table S1 and Supplementary Fig. S2). Immunoglobulin genes were similarly represented in other studies of genes that increase expression in tissues from aging animals, including lung, liver, and muscle [16, 23, 24]. The strong signature for antibody production in our analysis of lung tissue is consistent with a higher level CD19^+^ B cells seen in aged mice [13]. The antibodies encoded by these genes were not, however, contributing to anti-influenza responses since measurable hemagglutination inhibition titers were delayed in the time series of aged animals [13]. The specificity of these antibodies remains to be determined.

The strong immunoglobulin signature is consistent with the idea that B cells and immunoglobulins contribute to the aging process [25]. A unique set of B cells, termed age-associated B cells, are found in aged mice, particularly female mice such as those used in our experiments [26, 27]. B cell markers that include CD19 and CD21 localized to the ‘grey’ module of non-connectivity using WGCNA. The expression data, therefore, cannot discriminate among B cell populations, including age-associated B cells, contributing to the immunoglobulin signature. Further immunologic study of the B cell subsets in the lungs of aged animals is therefore warranted.

Despite the substantial differences between adult and aged mice, both expressed a subset of genes immediately after infection and later in the time series. GO analysis indicated the early response was enriched for antioxidant genes, which is consistent with prior knowledge of influenza: redox plays a complex role in viral infections, including influenza, and it has been suggested antioxidants could be a therapeutic target [28–30]. It was of particular interest that chaperonin genes were found in this subset of genes since influenza relies on cellular chaperonins to sustain the fitness of “biophysically defective” adaptive mutations [31]. Conversely, both cohorts of animals demonstrated strong CD8 T cell signatures later after infection. The kinetics of the CD8 T cell response in our study, peaking at day 9, was similar to that observed in another transcriptome analysis where the peak response was on day 8 [17]. T lymphocyte responses are central to the recovery from influenza infection [32], and our data support a model where CD8 cells aid in the resolution of infection in both age cohorts. Our dataset does not permit distinguishing the effects of infection from the stress of anesthesia or application of fluid into the lung at extremely early time points (< 24 h) [33]. Genes in the ‘brown2’ module may be responding to the infection, the experimental procedure per se, or both. The similar transcriptome alterations seen in ‘brown2’, along with ‘purple’ and ‘mediumpurple3’, however, illustrate that there are shared responses between the two age groups. Therefore, highly conserved, age-independent transcriptional changes were associated with the response to influenza even though there were substantial gene expression differences between the age groups.

In addition to the above analyses, the expression data in this manuscript was analyzed for an explanation of an immunologic paradox observed in our prior study. Peak viral loads in adult mice were higher than those seen in aged mice despite the superior anti-viral response observed in the younger animals [13]. The proposed mechanism involved lower cell proliferation and replicative senescence in older animals at baseline [13]. In support of this, the ‘cyan’ module that was associated with adult mice contained TERC, a component of telomerase that would antagonize senescence. Findings from the ‘yellow’ module appear to contradict this hypothesis. Strong signals for cell proliferation were found in the lungs of both animals after infection, but expression of these genes accelerated at the end of the time series in the aged animals (Supplementary Fig. S2). The gene encoding the proinflammatory cytokine IL-18 was also found in ‘yellow’. While the etiology of the elevated viral load in adult animals remains unclear, the combination of elevated cell cycle genes and *Il18* may contribute to viral persistence in the aged mice at the end of the time series [34].

## Conclusions

We have investigated the impact of aging on host defenses against influenza infection in the mouse model. In addition to validating our previous observations of immunologic alterations, the current work refines our prior study of aging with greater granularity by evaluating the transcriptome. Among our findings, we identify substantial differences in immunoglobulin gene expression and B cell signals associated with aging, which may be harnessed for biomarker analysis and studied as a possible mechanism that contributes to inflammaging in the lung.

## Materials and Methods

### Experimental design

In this study, we measured gene expression changes in mice of different ages that were infected with influenza. We used an experimental design similar to one that we conducted previously [13], except that fewer time points were sampled in the current work. The time points selected for this study allowed us to capture the initial transcription response to influenza infection along with any age-specific differences over time. The experimental design included adult (12–16 weeks) and aged (72–76 weeks) female BALB/c mice (Harlan-Sprague, Indianapolis, IN, USA) at the time points: 0 days (no infection), 1, 3, 5, 7, and 9 days post-infection. Each age and time point treatment were represented by three biological replicates (two ages x six time points x three biological replicates = 36 samples).

### Infections and sample collection

The mouse adapted influenza virus A/Puerto Rico/8/34 (H1N1) (A/PR8) was grown in MDCK cells as previously described [35]. A master stock of A/PR8 was prepared, aliquoted (100 µl/aliquot) and stored (-80 °C) until usage. A single virus aliquot was used to infect a set of animals on a determined day; the unused virus sample was discarded appropriately. Aliquots were evaluated periodically (plaque assay and HA content) to confirm stability and infectivity of the virus.

For infections, mice were anesthetized (ketamine and xylazine mix) and intranasally instilled with 50 μl of PBS containing 50–100 pfu of PR8. After infection, all animals were monitored daily for morbidity (weight loss and sickness score) and mortality, as described in [13]. All the protocols used were approved by the IACUC of the University of Pittsburgh.

To harvest lung tissue for RNA analysis, the lungs were first exposed by opening the chest cavity. Blood from the lungs was removed by irrigating the heart’s right ventricle with ice-cold PBS, 5–8 ml, until lungs became white–pink. The left lung was removed, snap-frozen, and stored at -80 °C until processing for microarray analysis.

### Tissue homogenization and RNA isolation

Whole mouse lungs were homogenized in 2 ml MagNA lyser Green Bead tubes (Roche Applied Science) with 1 mL TRI-reagent (Molecular Research Center, Inc.) at 6000 rpm for 1 min. Homogenates were transferred to 1.5 mL RNase-free safe-lock tubes (Eppendorf) containing 1 mL of TRI-Reagent (Molecular Research Center). For RNA isolation with TRI-Reagent, the manufacturer’s protocol for phase separation and RNA precipitation were followed except that samples were placed at -80 °C overnight after adding isopropanol.

To complete the isolation, tubes were thawed on ice, RNA was pelleted by centrifugation (12,000 rpm, 45 min, 4 °C), and pellets were washed twice with cold 75% RNase-free ethanol (1 mL, 12,000 rpm, 10 min, 4 °C). After removing the ethanol wash, RNA pellets were dried for 3–5 min on the bench top, and then resuspended in 50 µL RNase-free water. RNA was stored at -80 °C until microarray labeling. RNA integrity was evaluated using the Agilent RNA 6000 Nano Kit and 2100 BioAnalyzer according to the product handbooks. RNA quantity was measured using a DU 800 UV/Vis Spectrophotometer (Beckman Coulter).

### RNA labeling and microarray hybridization, and data preprocessing

Five hundred ng of total lung RNA was reverse transcribed to cDNA and processed to cyanine-3 labeled cRNA using the one-color Quick Amp Labeling Kit (Agilent) according to the product handbook. Labeled cRNA samples were purified using the RNeasy mini kit (QIAGEN). Cy-3 integration and cRNA amplification were assessed using spectrophotometry, and samples with approximately 10 pmol Cy3/ug cRNA integration and greater than 5 µg total cRNA yield were hybridized to arrays. Labeled cRNA (1.65 µg) was hybridized to Whole Mouse Genome 4 × 44 K microarrays (Agilent) following the manufacturer’s recommendations. Array chambers were placed in a rotating hybridization oven (10 rpm, 65 °C) for 17 h. After hybridization, arrays were washed using pre-warmed (37 °C) gene expression wash buffer 2 (Agilent) and scanned immediately in an Agilent G2565 microarray scanner.

Background correction was performed using the *limma* R package [19]: [backgroundCorrect command]: method = "normexp", offset = 1. We further normalized the samples between arrays using normalizeBetweenArrays and the ‘quantile’ option, implemented in *limma*. We selected one probe per gene that corresponded with the maximum mean expression value across the 34 samples. Three replicates were used per age class x timepoint condition except Day 3, which contained two replicates in both adult and aged samples. These two outlier samples were highlighted using the adjacency matrix and flashClust functions in WGCNA, followed by visual inspection of the plotDendroAndColors dendrogram. Control probes and probes with no gene annotation were excluded from the data set. The pre-processed expression data set contained features that queried 27,962 genes.

Principal components were calculated using the complete 34-sample data set and the prcomp function in the stats R package.

### Weighted Gene Co-expression Network Analysis (WGCNA)

Transcript expression values were log_2_-transformed prior to module assignment. We empirically estimated the soft pick threshold (power) at 16 for a signed network with a R^2^ value ~ 0.9 [18]. The scale-free topological overlap matrix (the basis of the module assignment) was constructed using the following parameters: maxBlockSize = 1000, TOMType = "signed", randomseed = 12,345, power = 16, minModuleSize = 30, mergeCutHeight = 0, numericLabels = TRUE, saveTOMs = TRUE, pamRespectsDendro = FALSE. Using no module merging allowed very subtle differences between modules to be separated into discrete groups of co-expressed genes. Modules consistent with age-associated and age x time interactions could then be grouped retrospectively, if necessary. WGCNA assigns module identities as an arbitrary color and in the remaining text, we report the colors that were assigned.

To explore possible associations between modules and experimental age and time treatments in WGCNA, mouse age was coded as a binary trait (adult = 0, aged = 1) and each time treatment was coded as 1 or 0 as appropriate. For example, all arrays from day 0 would be scored as ‘1’ for day 0, and ‘0’ for all other days. Likewise, all arrays from day 1 would be scored as ‘1’ for day 1 treatment and ‘0’ for all other days. All days were scored in the same way so correlations between the binary classifier could be tested.

To formally test age x timepoint interactions, we used module eigengenes determined by WGCNA, as a proxy of expression. Models were constructed and fit in the limma R package [19]. The model was constructed to accommodate the replicate samples at each time point. We fit separate curves (natural regression splines) for the adult and elderly samples and an appropriate degree-of-freedom (df = 4) as per the limma reference manual recommendations. We then formally tested whether module eigengenes across samples were associated with age effects, the time curve (spline), or their higher order interaction. The model was fit using lmFit, followed by ebayes moderation in limma. All model coefficients were simultaneously estimated and a table of the top ranked module list from the lmFit were tabulated using topTable and the “BH” method [36] to control the False Discovery Rate. Modules with FDR < 0.05 are shown in Supplementary Fig. S2. An FDR < 0.05 is considered nominally significant.

Throughout the data visualizations in heatmaps and interaction plots, expression data were scaled to be zero centered for visual clarity.

## Supplementary information

**Additional file 1:** **Fig. S1 **Module-Trait relationship between module eigengenes and experimental traits, including mouse age class and days post infection. Rows represent modules and columns represent experimental traits: age (aged versus adult), ‘Day_of_Infect’ which represents individual days after infection (Day 0 versus the other days, Day 1 versus the other days, etc.), and the numerical day post-infection (0,1,3,5,7,9). Each cell of the matrix contains two values. The upper value is the correlation coefficient (rho, r) and the lower value is the p-value of the correlation between the module eigengene for each module and the trait in question (column). The Red-White-Green heat component represents the scale from *r *= 1.0 to *r *= -1.0

**Additional file 2:** **Fig. S2** Characteristics of statistically supported modules that demonstrate various age, expression curve (spline), and age x curve interaction effects as determined by formal differential expression modeling. Each row represents a separate module and contains the respective heatmap-barplot combination figure (left plot), the interaction plot (top 30 representative genes; black = adult, module color = aged) (middle plot), and a gene ontology (GO) summary for the complete module membership (right table). The heatmap and barplot (far left of each row) show the scaled gene expression and average eigengene expression for each sample, respectively, which are ordered by increasing timepoints from left to right in each age class (adult: left; and aged: right). The interaction plot (center panel of each row) shows the behavior of the top 30 genes by their module membership. Each line is a separate gene and the same genes are shown for both age classes. GO ‘Process’, ‘Function’ and ‘Compartment’ categories are reported for the four most significant ontologies in each category (far right panel of each row). The 15 significant modules with FDR<0.05 are shown and the bottom module is an additional immune related module with FDR=0.057 (‘mediumpurple3’)

**Additional file 3:** **Fig. S3 **Line plots of nine cytokines and chemokines found in both the present study and our previous study (Toapanta and Ross (2009)). Each line plot shows scaled gene expression over time for each of the individual genes in adult (black lines) and aged mice (non-black) lines. Genes with a greenyellow line for the aged animals (*Il1b*, *Ifnb1*, *Il6*, *Cxcl1*), were clustered in the ‘greenyellow’ module. Likewise, genes with a green line for aged animals (*Ccl4*, *Ccl5*, *Ccl2*, *Ifng*, *Tnf*) were clustered in the ‘green’ module (see main text for details). Overall, adult mice showed a faster gene expression response than the aged mice

**Additional file 4:** **Table S1**

**Additional file 5:** **Table S2**

## Data Availability

RNA expression data and sample metadata are deposited in the GEO data repository under accession: GSE197596. The files will be publicly available upon manuscript acceptance or 6 months after submission, whichever is first. A script to recapitulate the analysis is available upon request.
