# Supplementary material for: Altered transcriptional responses in the lungs of aged mice after influenza infection
Source: Immun Ageing. 2022 Jun 1;19:27. doi: 10.1186/s12979-022-00286-9 (PMC9158162; doi:10.1186/s12979-022-00286-9)

Module-trait relationships (Hernandez et al)

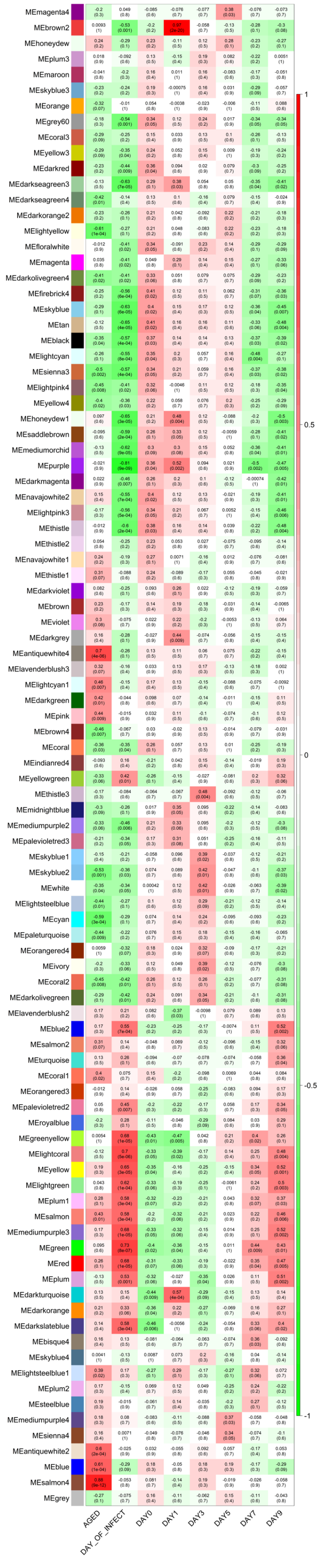

Supplement: Supplementary file 1 — Additional file 1: Fig. S1 Module-Trait relationship between module eigengenes and experimental traits, including mouse age class and days post infection. Rows represent modules and columns represent experimental traits: age (aged versus adult), ‘Day_of_Infect’ which represents individual days after infection (Day 0 versus the other days, Day 1 versus the other days, etc.), and the numerical day post-infection (0,1,3,5,7,9). Each cell of the matrix contains two values. The upper value is the correlation coefficient (rho, r) and the lower value is the p-value of the correlation between the module eigengene for each module and the trait in question (column). The Red-White-Green heat component represents the scale from r = 1.0 to r = -1.0 [file 12979_2022_286_MOESM1_ESM.pdf]
