# Supplementary material for: Altered transcriptional responses in the lungs of aged mice after influenza infection
Source: Immun Ageing. 2022 Jun 1;19:27. doi: 10.1186/s12979-022-00286-9 (PMC9158162; doi:10.1186/s12979-022-00286-9)
Supplement: Supplementary file 2 — Additional file 2: Fig. S2 Characteristics of statistically supported modules that demonstrate various age, expression curve (spline), and age x curve interaction effects as determined by formal differential expression modeling. Each row represents a separate module and contains the respective heatmap-barplot combination figure (left plot), the interaction plot (top 30 representative genes; black = adult, module color = aged) (middle plot), and a gene ontology (GO) summary for the complete module membership (right table). The heatmap and barplot (far left of each row) show the scaled gene expression and average eigengene expression for each sample, respectively, which are ordered by increasing timepoints from left to right in each age class (adult: left; and aged: right). The interaction plot (center panel of each row) shows the behavior of the top 30 genes by their module membership. Each line is a separate gene and the same genes are shown for both age classes. GO ‘Process’, ‘Function’ and ‘Compartment’ categories are reported for the four most significant ontologies in each category (far right panel of each row). The 15 significant modules with FDR<0.05 are shown and the bottom module is an additional immune related module with FDR=0.057 (‘mediumpurple3’) [file 12979_2022_286_MOESM2_ESM.pdf]

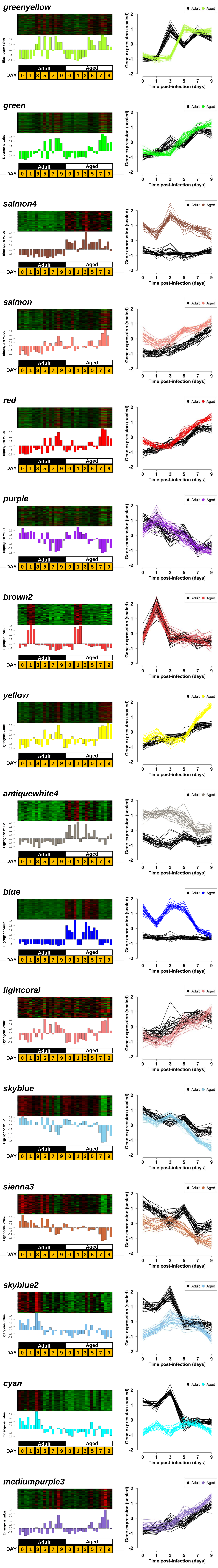

greenyellow	GO term	Description	FDR
Process	GO:0051607	defense response to virus	3.83E-36
	GO:0009615	response to virus	6.43E-36
	GO:0051707	response to other organism	8.83E-29
	GO:0043207	response to external biotic stimulus	5.51E-28
Function	GO:0003725	double-stranded RNA binding	3.78E-08
	GO:0001730	2'-5'-oligoadenylate synthetase activity	1.18E-03
	GO:0042802	identical protein binding	1.49E-03
	GO:0070566	adenylyltransferase activity	4.04E-03
Compartment	GO:0044444	cytoplasmic part	1.01E-02
	GO:0044424	intracellular part	2.48E-02
	GO:0005829	cytosol	3.60E-02
	GO:0005737	cytoplasm	8.55E-02

green	GO term	Description	FDR
Process	GO:0002376	immune system process	2.19E-27
	GO:0006952	defense response	3.85E-20
	GO:0006955	immune response	5.26E-20
	GO:0043207	response to external biotic stimulus	4.06E-18
Function	GO:0008009	chemokine activity	2.54E-03
	GO:0005126	cytokine receptor binding	3.02E-03
	GO:0001614	purinergic nucleotide receptor activity	2.05E-03
	GO:0016502	nucleotide receptor activity	1.54E-03
Compartment	GO:0016020	membrane	5.10E-08
	GO:0009897	external side of plasma membrane	3.96E-08
	GO:0098552	side of membrane	4.39E-07
	GO:0044425	membrane part	1.93E-03

salmon4	GO term	Description	FDR
Process	GO:0002377	immunoglobulin production	8.64E-14
	GO:0002440	production of molecular mediator of immune response	2.15E-12
	GO:0006955	immune response	4.99E-09
	GO:0002376	immune system process	7.02E-06
Function	GO:0034987	immunoglobulin receptor binding	1.69E-05
	GO:0003823	antigen binding	3.09E-03
Compartment	GO:0044421	extracellular region part	1.61E-07
	GO:0042571	immunoglobulin complex, circulating	3.34E-07
	GO:0019814	immunoglobulin complex	1.26E-06
	GO:0005615	extracellular space	5.70E-06

salmon	GO term	Description	FDR
Process	GO:0002694	regulation of leukocyte activation	3.68E-16
	GO:0051249	regulation of lymphocyte activation	3.20E-16
	GO:0050865	regulation of cell activation	2.29E-15
	GO:0002376	immune system process	4.24E-14
Function	GO:0023026	MHC class II protein complex binding	3.12E-05
	GO:0023023	MHC protein complex binding	5.59E-04
	GO:0001784	phosphotyrosine residue binding	3.40E-02
	GO:0051219	phosphoprotein binding	6.62E-02
Compartment	GO:0042613	MHC class II protein complex	1.12E-11
	GO:0042611	MHC protein complex	4.23E-08
	GO:0044464	cell part	2.98E-04
	GO:0044459	plasma membrane part	2.89E-03

red	GO term	Description	FDR
Process	GO:0002376	immune system process	2.99E-26
	GO:0002682	regulation of immune system process	4.09E-20
	GO:0002684	positive regulation of immune system process	2.54E-16
	GO:0045321	leukocyte activation	4.39E-15
Function	GO:0005515	protein binding	3.40E-09
	GO:0003779	actin binding	7.05E-08
	GO:0051015	actin filament binding	1.13E-06
	GO:0005488	binding	2.78E-04
Compartment	GO:0016020	membrane	4.92E-16
	GO:0005886	plasma membrane	1.70E-13
	GO:0044459	plasma membrane part	1.50E-07
	GO:0031982	vesicle	4.22E-06

purple	GO term	Description	FDR
Process	GO:0002483	antigen processing and presentation of endogenous peptide antigen	2.59E-12
	GO:0019885	antigen processing and presentation of endogenous peptide antigen via MHC class I	1.30E-12
	GO:0019883	antigen processing and presentation of endogenous antigen	3.74E-12
	GO:0002474	antigen processing and presentation of peptide antigen via MHC class I	2.30E-11
Function	GO:0042605	peptide antigen binding	6.59E-10
	GO:0046977	TAP binding	3.46E-08
	GO:0062061	TAP complex binding	9.68E-08
	GO:0042610	CD8 receptor binding	8.50E-07
Compartment	GO:0044217	other organism part	1.71E-09
	GO:0033643	host cell part	1.01E-09
	GO:0042824	MHC class I peptide loading complex	1.47E-09
	GO:0042612	MHC class I protein complex	2.24E-09

brown2	GO term	Description	FDR
Process	GO:0051186	cofactor metabolic process	3.02E-05
	GO:0044281	small molecule metabolic process	8.12E-03
	GO:0006749	glutathione metabolic process	6.30E-03
	GO:0019852	L-ascorbic acid metabolic process	4.85E-03
Function	GO:0016491	oxidoreductase activity	3.21E-03
	GO:1900750	oligopeptide binding	3.67E-02
	GO:0043295	glutathione binding	2.44E-02
	GO:0003824	catalytic activity	2.30E-02
Compartment	GO:0044444	cytoplasmic part	2.87E-01

yellow	GO term	Description	FDR
Process	GO:0007049	cell cycle	3.02E-57
	GO:0022402	cell cycle process	6.46E-49
	GO:0051301	cell division	2.58E-45
	GO:1903047	mitotic cell cycle process	9.85E-44
Function	GO:0005524	ATP binding	6.01E-08
	GO:0008017	microtubule binding	6.99E-08
	GO:0032559	adenyl ribonucleotide binding	4.84E-08
	GO:0030554	adenyl nucleotide binding	5.45E-08
Compartment	GO:0005694	chromosome	6.74E-40
	GO:0000775	chromosome, centromeric region	5.16E-39
	GO:0098687	chromosomal region	5.85E-37
	GO:0044427	chromosomal part	9.32E-37

antiquewhite4	GO term	Description	FDR
Process	GO:0007606	sensory perception of chemical stimulus	3.71E-22
	GO:0007608	sensory perception of smell	8.15E-22
	GO:0007600	sensory perception	1.35E-18
	GO:0007186	G protein-coupled receptor signaling pathway	1.10E-16
Function	GO:0004984	olfactory receptor activity	2.54E-22
	GO:0004888	transmembrane signaling receptor activity	1.54E-20
	GO:0038023	signaling receptor activity	2.91E-19
	GO:0060089	molecular transducer activity	4.44E-19
Compartment	GO:0016021	integral component of membrane	1.96E-09
	GO:0031224	intrinsic component of membrane	3.14E-09
	GO:0044425	membrane part	6.34E-08

blue	GO term	Description	FDR
Process	GO:0050877	nervous system process	1.30E-18
	GO:0007608	sensory perception of smell	8.38E-17
	GO:0007186	G protein-coupled receptor signaling pathway	5.67E-17
	GO:0007606	sensory perception of chemical stimulus	1.19E-16
Function	GO:0004888	transmembrane signaling receptor activity	3.80E-26
	GO:0038023	signaling receptor activity	1.19E-24
	GO:0060089	molecular transducer activity	3.87E-24
	GO:0004984	olfactory receptor activity	2.53E-17
Compartment	GO:0031224	intrinsic component of membrane	1.05E-09
	GO:0016021	integral component of membrane	1.59E-09
	GO:0044425	membrane part	2.38E-06
	GO:0031226	intrinsic component of plasma membrane	2.50E-04

lightcoral	GO term	Description	FDR
Process	GO:0044238	primary metabolic process	2.71E-01
	GO:0008152	metabolic process	3.10E-01
	GO:0009894	regulation of catabolic process	2.08E-01
	GO:0031329	regulation of cellular catabolic process	1.77E-01
Function	GO:0004190	aspartic-type endopeptidase activity	5.61E-01
	GO:0070001	aspartic-type peptidase activity	3.12E-01
	GO:0140096	catalytic activity, acting on a protein	7.81E-01
Compartment	GO:0043231	intracellular membrane-bounded organelle	1.36E-01
	GO:0044815	DNA packaging complex	7.43E-02
	GO:0043227	membrane-bounded organelle	1.67E-01
	GO:0044437	vacuolar part	3.08E-01

skyblue	GO term	Description	FDR
Process	GO:0003012	muscle system process	2.74E-01
	GO:0006941	striated muscle contraction	1.51E-01
	GO:0006936	muscle contraction	1.14E-01
	GO:0003009	skeletal muscle contraction	1.62E-01
Function	GO:0005509	calcium ion binding	3.44E-04
	GO:0043167	ion binding	3.91E-03
	GO:0046872	metal ion binding	4.21E-02
	GO:0043169	cation binding	6.01E-02
Compartment	GO:0044449	contractile fiber part	5.53E-05
	GO:1990584	cardiac Troponin complex	1.54E-01
	GO:0033017	sarcoplasmic reticulum membrane	3.45E-01
	GO:0031672	A band	2.59E-01

sienna3	GO term	Description	FDR
Process	GO:0007155	cell adhesion	1.00E+00
	GO:0070458	cellular detoxification of nitrogen compound	1.00E+00
	GO:0051410	detoxification of nitrogen compound	1.00E+00
	GO:0022610	biological adhesion	1.00E+00
Function	GO:0005201	extracellular matrix structural constituent	1.39E-02
Compartment	GO:0062023	collagen-containing extracellular matrix	9.91E-01

skyblue2	GO term	Description	FDR
Process			
Function	GO:0038023	signaling receptor activity	9.90E-01
	GO:0060089	molecular transducer activity	6.19E-01
Compartment			

cyan	GO term	Description	FDR
Process	GO:0034287	detection of monosaccharide stimulus	1.00E+00
	GO:0051594	detection of glucose	7.18E-01
	GO:0009732	detection of hexose stimulus	4.79E-01
	GO:0009730	detection of carbohydrate stimulus	3.59E-01
Function	GO:0038023	signaling receptor activity	2.80E-01
	GO:0060089	molecular transducer activity	2.17E-01
	GO:0004888	transmembrane signaling receptor activity	3.55E-01
	GO:0004930	G protein-coupled receptor activity	3.95E-01
Compartment	GO:0031526	brush border membrane	1.83E-01

mediumpurple3	GO term	Description	FDR
Process	GO:0002376	immune system process	4.14E-15
	GO:0002682	regulation of immune system process	8.62E-15
	GO:0002684	positive regulation of immune system process	6.93E-12
	GO:0050776	regulation of immune response	6.88E-11
Function	GO:0016811	hydrolase activity, acting on carbon-nitrogen (but not peptide) bonds, in linear amides	5.89E-04
	GO:0016810	hydrolase activity, acting on carbon-nitrogen (but not peptide) bonds	6.74E-04
	GO:0004896	cytokine receptor activity	2.08E-01
	GO:0016493	C-C chemokine receptor activity	2.72E-01
Compartment	GO:0042101	T cell receptor complex	1.80E-07
	GO:0009897	external side of plasma membrane	3.90E-07
	GO:0098552	side of membrane	3.50E-07
	GO:0001772	immunological synapse	2.65E-05
