# Supplementary figures and images for: Altered transcriptional responses in the lungs of aged mice after influenza infection
Source: Immun Ageing. 2022 Jun 1;19:27. doi: 10.1186/s12979-022-00286-9 (PMC9158162; doi:10.1186/s12979-022-00286-9)

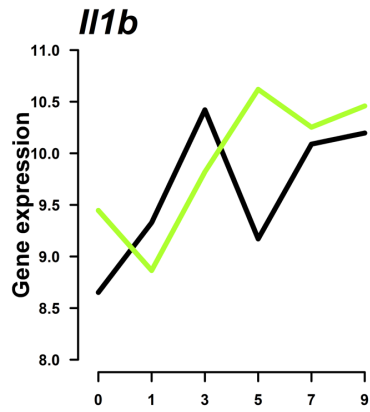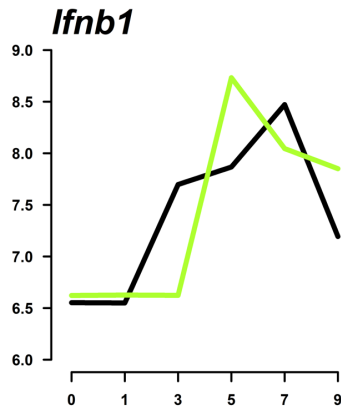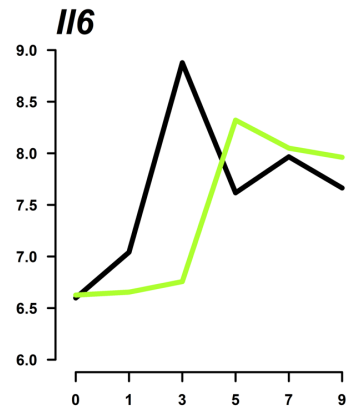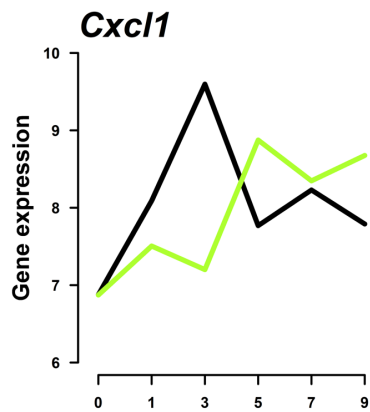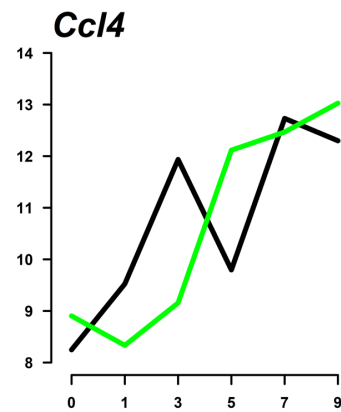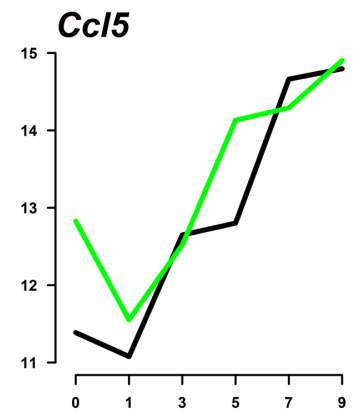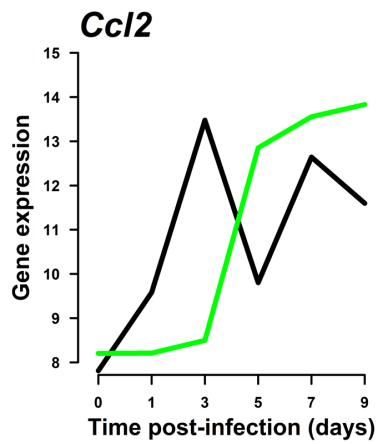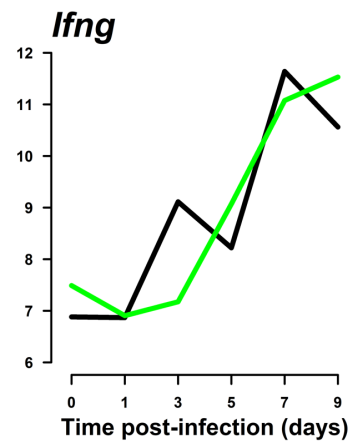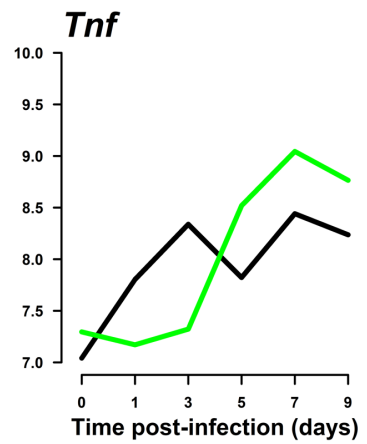

Supplement: Supplementary file 3 — Additional file 3: Fig. S3 Line plots of nine cytokines and chemokines found in both the present study and our previous study (Toapanta and Ross (2009)). Each line plot shows scaled gene expression over time for each of the individual genes in adult (black lines) and aged mice (non-black) lines. Genes with a greenyellow line for the aged animals (Il1b, Ifnb1, Il6, Cxcl1), were clustered in the ‘greenyellow’ module. Likewise, genes with a green line for aged animals (Ccl4, Ccl5, Ccl2, Ifng, Tnf) were clustered in the ‘green’ module (see main text for details). Overall, adult mice showed a faster gene expression response than the aged mice [file 12979_2022_286_MOESM3_ESM.pdf]
